# Extraordinary Results in the Treatment of Diphtheria

**Published:** 1901-12

**Authors:** Francis E. Fronczak

**Affiliations:** Buffalo, N.Y.


### Extraordinary Results in the Treatment of Diphtheria.

By FRANCIS E. FRONCZAK, M. D., Buffalo, N. Y.

The following is a report on two cases of diphtheria in which I used antidiphtheritic serum with extraordinary results:

The first case was that of a boy one and a half years of age. I was called to visit the child on the third day of the disease and found him suffering from diphtheria, the tonsils being very much enlarged and swollen, and membrane present in large quantities. I wished to intubate, but as the family objected, I administered 2,000 units of the antidiphtheritic serum of Messrs. Parke, Davis & Co., following with 1,500 units in the evening of the next day. Three days afterward he was out of all danger, and a culture made on the tenth day of the disease was reported by the Health Department to contain no Klebs-Löffler bacilli.

The second case was that of a girl, 6 years old. I was called on the fifth day of the disease, and found the patient comatose. Here again it seemed to be that intubation was the only means of saving the girl's life, but the family being opposed to it, I gave her 3,000 units of antidiphtheritic serum (P., D. & Co.) following two days latter with 2,000 units more. Six days after the first injection the girl was up and around the house. On the thirteenth day of the disease a culture sent to the Health Department was reported on negatively.

The usual applications of antiseptics were employed in addition to the treatment with the antidiphtheritic serum, yet I do not hesitate to say that the serum was the means of saving life in both cases. I have also used the same brand of serum in eight other cases during the last two months, and thus far I have had no fatalities.

